# Short-Term Preliminary Evaluation of Suicide Following the 2024 Noto Peninsula Earthquake in Japan Using Time Series Analysis

**DOI:** 10.1027/0227-5910/a001003

**Published:** 2025-04-30

**Authors:** Takahiro Arai

**Affiliations:** ^1^ School of Management and Information Sciences, Tama University, Tokyo, Japan; ^2^ Graduate School of Health Management, Keio University, Kanagawa, Japan

**Keywords:** suicide, Japan, earthquake, pulling together effect, time series analysis

## Abstract

**Abstract:** *Background*: The impact of earthquakes on mental health is profound. *Aim*: This study examines the short-term impact of the Noto Peninsula earthquake (magnitude 7.6) in Japan in January 2024 on the number of suicides and investigates the existence of the *pulling together effect* or *honeymoon phase* in suicide trends. *Method*: Suicide data from disaster-affected areas in Ishikawa Prefecture from January 2017 to June 2024 were analyzed using Poisson regression and prophet models. *Results*: Both models identified fewer suicides than predicted for 3 consecutive months (March–May 2024). This trend was observed even when the affected areas were subdivided into multiple regions. *Limitations*: Economic factors and data on suicide attempts or mental disorders were not included in the analysis. *Conclusion*: This study provides evidence supporting the pulling together effect or honeymoon phase, suggesting that increased community support following a disaster temporarily reduces suicide risk.

The impact of earthquakes on people's mental health is profoundly serious. Following an earthquake, individuals suffer not only direct physical harm but also psychological and social consequences. Experiences such as home collapses, disrupted lifelines, and the loss of family and friends can lead to mental health issues, including posttraumatic stress disorder (PTSD), depression, and anxiety disorders (Fan et al., 2011; Goenjian et al., 2000; Qu et al., 2012; Soqia et al., 2023; Ticehurst et al., 1996; Zhang et al., 2011). Additionally, prolonged displacement and stress from changes in living conditions further exacerbate mental health risks (Cénat et al., 2021; Salcioglu et al., 2007).

Postdisaster mental health care is a crucial public health issue that requires appropriate measures. However, the actual state of postdisaster mental health and the effectiveness of interventions remain unclear (Fitzpatrick & Spialek, 2020; Krug et al., 1998). In particular, fluctuations in suicide rates are an important indicator for assessing postdisaster mental health. A detailed study of these fluctuations can provide essential data for the implementation of appropriate mental health support measures.

Following the Kobe earthquake (magnitude 7.3), suicide rates significantly declined in the 2 years postdisaster, with Kobe City experiencing its lowest suicide rate since World War II (Nishio et al., 2009; Shioiri et al., 1999). This suggests that suicide rates may decrease in the short term immediately after an earthquake. The Great East Japan Earthquake (magnitude 9.0) had a significant negative impact on mental health due to the combined effects of the earthquake, tsunami, and nuclear power plant accident (Ando et al., 2017; Harada et al., 2015; Yamashita & Shigemura, 2013; Yokoyama et al., 2014). However, in the 2 years following the disaster, suicide rates in the affected areas decreased compared to 2010, with a slight decrease observed in Japan as a whole (Ohto et al., 2015). This trend was also noted in evacuated areas (Orui et al., 2018).

The phenomenon of lower suicide rates immediately after major catastrophes is known as the *pulling together effect* or *honeymoon phase*, which temporarily strengthens feelings of support and solidarity (Kõlves et al., 2013). It has been observed that during the initial postearthquake phase (1 month to 2 years), suicide rates decrease due to strengthened community solidarity and support but then increase as support diminishes and the harsh reality sets in (Jafari et al., 2020; Safarpour et al., 2020).

On 1 January 2024, a magnitude 7.6 earthquake struck the Noto Peninsula in Ishikawa Prefecture, Japan, resulting in 281 deaths, destroying 127,334 homes, and disrupting essential lifelines such as water and electricity (Cabinet Office, 2024; Ministry of Land, Infrastructure, Transport and Tourism, Japan Meteorological Agency, 2024). Despite 6 months having passed at the time we conducted this study, the effects persisted, raising concerns about the impact on suicides. According to previous hypotheses, suicide risk was expected to decrease immediately after the earthquake; however, empirical evidence remained limited due to the recency of the event. This study aimed to analyze the variation in suicides in the 6 months following the Noto Peninsula earthquake and to test whether the honeymoon phase was observed.

## Methods

### Data Collection

The number of suicide deaths in Ishikawa Prefecture from January 2017 to June 2024 was obtained from open data from the Ministry of Health, Labour, and Welfare. The dataset consisted of 90 months, with January 2017 to December 2023 as the study period and January 2024 to June 2024 as the test period. Population data as of 1 October in each year were used, as population estimates are published on 1 October each year in Japan.

The 2024 Noto Peninsula earthquake occurred at 16:10 on 1 January 2024. The epicenter was at 37°49′ north latitude and 137°27′ east longitude (see Figure E1 in Electronic Supplementary Material 1 [ESM 1]), with a magnitude of M 7.6. The disaster area was defined as the Noto region, consisting of 13 municipalities: the cities of Nanao, Wajima, Suzu, Hakui, Kahoku, Tsubata, Uchinada, Shika, Hodatsushimizu, Nakanoto, Anamizu, Noto, and Kanazawa (Table E1 in ESM 1). Data on short-term casualties and totally destroyed houses in disaster areas are presented in Table E1 (Cabinet Office, 2024). Kanazawa City suffered only minor damage; however, it was included in the disaster area because it is the prefectural capital and has strong economic links with the 12 disaster areas, as well as economic damage as a tourist destination.

### Statistical Analysis

In this observational study, the impact of the 2024 Noto Peninsula earthquake on the number of suicides was assessed by excess deaths using time series analysis.

Using Poisson regression models to analyze excess suicides is a common method (Moksony & Hegedűs, 2014; Sakamoto et al., 2024). Poisson regression models are suitable for predicting count data and assume that the dependent variable follows a Poisson distribution. In the Poisson regression model, the monthly number of suicides was used as the outcome and annual continuous variables, monthly dummies, and population were included as explanatory variables as offset terms.

$$y_{t}∼\text{Poisson}\left(\mu_{t}\right)$$

$$\log\left(\mu_{t}\right)=\beta_{0}+\beta_{Y}\cdot \text{Yea}r_{t}+\overset{12}{\underset{M=1}{\sum }}\beta_{M}\cdot \text{Mont}h_{t}+\text{offset}\left(\log\left({\text{Population}}_{t}\right)\right).$$

Here, ${\text{Year}}_{t}$ is the year at *t*, $\text{Mont}h_{t}$ is the month at *t*, $\text{Populatio}n_{t}$ is the population at *t*. The parameters $\beta_{0}$, $\beta_{Y}$, and $\beta_{M}$ are the coefficients to be estimated.

This allows for temporal and demographic changes affecting the number of suicides to be taken into account.

To evaluate the performance of different time series forecasting models, both the Poisson regression model and the prophet model were used. Prophet models are effective in capturing seasonality and trend fluctuations, and robust results can be expected by assessing how models with different characteristics perform. Prophet models enable the identification of annual cyclical patterns in the data (Kirpich et al., 2022; Taylor & Letham, 2018).

$$y_{t}=g\left(t\right)+s\left(t\right)+\epsilon_{t}$$

Here, $y_{t}$ represents the observed value at time *t*, g(t) is the trend component, s(t) is the seasonal component, and $\epsilon_{t}$ is the residual. The prophet model predicts by considering both long-term trends and seasonal variations in the data. Since this model used monthly data, holiday effects were not included; however, annual seasonality and long-term trends were incorporated.

All statistical analyses were performed using R software (version 4.3.3), and the significance level was set to 5%. Ethical review was not required for this study as it utilized publicly available data. The data were anonymized and publicly available, ensuring that individuals could not be identified.

## Results

The annual average number of suicides from 2017 to 2023 was 10.75, 8.25, 9.75, 9.25, 7.25, 10.0, and 10.42. In the first half of 2024, the average monthly number of suicides was 6.67. A total of 40 suicides were reported in the first 6 months of 2024: 11 in January, 7 in February, 4 in March, 5 in April, 4 in May, and 9 in June.

**Figure 1:**
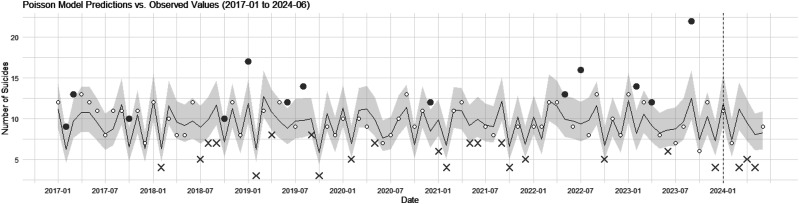
Comparison of predicted and observed suicides with confidence interval for 2024 using Poisson regression model. This figure displays the monthly observed number of suicides in disaster areas from January 2017 to June 2024, as well as the monthly predicted suicides. The black solid line indicates the predicted number of suicides, while the gray shaded area represents the 95% confidence interval. Months in which the observed number of suicides is outside the 95% confidence interval are marked. Excess suicides are indicated by filled bold circles, while fewer suicides than predicted are indicated by bold crosses.

The residual plots, QQ plots, and histograms from the Poisson regression model, along with the overdispersion test (see ESM 2), indicated that the model adequately fitted the data. Predicted values, confidence intervals, and measured values over the test period were plotted to identify months with excess or fewer suicides than predicted (Figure 1).

According to the Poisson regression model, January and February 2024 – the months of the earthquake – fell within the confidence interval, indicating neither excess nor fewer suicides than predicted. This trend was also observed in June. In March, the predicted value was 11.16 (CI: 8.60–14.48); in April, it was 9.47 (CI: 7.25–12.38); and in May, it was 8.06 (CI: 6.11–10.65), indicating fewer suicides than predicted across all 3 months.

**Figure 2:**
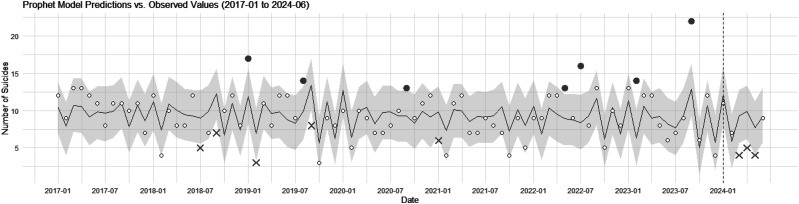
Comparison of predicted and observed suicides with confidence interval for 2024 using prophet model. This figure displays the monthly observed number of suicides in disaster areas from January 2017 to June 2024, as well as the monthly predicted suicides. The black solid line indicates the predicted number of suicides, while the gray shaded area represents the 95% confidence interval. Months in which the observed number of suicides is outside the 95% confidence interval are marked. Excess suicides are indicated by filled bold circles, while fewer suicides than predicted are indicated by bold crosses.

The prophet model showed that the actual number of suicides was within the confidence interval in January, February, and June 2024. However, in March, the predicted value was 9.31 (CI: 5.98–12.58), in April it was 9.88 (CI: 6.31–13.48), and in May it was 7.66 (CI: 3.98–10.95; Figure 2). This indicates that the number of suicides in March, April, and May was significantly below the lower bound of the confidence interval. Furthermore, the total predicted number of suicides from January to June 2024 was 54.01, whereas the actual number was 40, indicating that there were approximately 14 fewer suicides than predicted.

Root-mean-square error (RMSE), mean absolute error (MAE), and mean absolute percentage error (MAPE) were calculated to compare the performance of the Poisson regression and prophet models. The RMSE, MAE, and MAPE for the Poisson regression model were 2.57, 2.00, and 24.78%, respectively. In contrast, the RMSE, MAE, and MAPE for the prophet model were 2.79, 2.21, and 28.76%, respectively. These results demonstrate that the Poisson regression model has smaller prediction errors for all indicators compared to the prophet model. Therefore, the Poisson model was superior on all indicators.

In additional analyses using the prophet model for the Hokuriku region (both with and without Ishikawa Prefecture) and Japan (both with and without Ishikawa Prefecture), fewer suicides than predicted were observed in March 2024 when Ishikawa Prefecture was included in the analysis for Japan (see ESM 3). However, when Ishikawa Prefecture was excluded, no such decrease was observed in March. Additionally, fewer suicides than predicted were observed in the Hokuriku region, which includes Ishikawa Prefecture, in both March and May. In contrast, when Ishikawa Prefecture was excluded from the Hokuriku region, no fewer suicides than predicted were observed after the earthquake.

Additional analyses were conducted for the six areas that were directly affected by the earthquake (Nanao City, Wajima City, Suzu City, Shika Town, Anamizu Town, and Noto Town), as well as for 12 areas, which include these six areas plus Hakui City, Kahoku City, Tsubata Town, Uchinada Town, Hodatsushimizu Town, and Nakanoto Town (all municipalities listed in Table E1 in ESM 1 except for Kanazawa City). In addition, Kanazawa City, where economic damage was expected, and the area encompassing Kanazawa City and Kaga City were also included in the analysis, bringing the total to four areas. The results showed that fewer suicides than predicted were observed in April in the six directly affected areas, in April and May in the 12 areas, in March in Kanazawa City, and in March in the Kanazawa City and Kaga City area (see ESM 3).

## Discussion

In this study, the impact of the Noto Peninsula earthquake in 2024 on the number of suicides was assessed in the short term, focusing on the 6 months following the disaster.

The Poisson regression model and the prophet model identified fewer suicides than predicted for 3 consecutive months immediately after the earthquake – March, April, and May. Furthermore, at both the Hokuriku and national levels, fewer suicides than predicted were more frequently observed when Ishikawa Prefecture was included than when it was excluded. In addition, when the analysis was subdivided into the six directly affected areas, the 12 broader areas, Kanazawa City alone, and the combined area of Kanazawa City and Kaga City, fewer suicides than predicted were consistently observed across all four areas. This suggests that the number of suicides may have decreased significantly in the short period immediately after the disaster. The results of this analysis support the pulling together effect or honeymoon phase observed in previous studies, in which suicide rates decrease after the disaster by temporarily strengthening feelings of support and solidarity (Kõlves et al., 2013).

This reduction may also have been influenced by the activities of external support teams, such as the Disaster Psychiatry Assistance Teams (DPAT) and public health support teams. For instance, a total of 196 DPAT teams were dispatched to Ishikawa Prefecture. Additionally, between January 6 and May 30, 2024, a cumulative total of 9,434 public health nurses and 6,055 other professionals – including administrative staff, drivers, physicians, and pharmacists – from 42 prefectures contributed to the region's recovery efforts (Ministry of Health, Labour and Welfare, 2024a, 2024b).

Detailed comparisons of the mental health infrastructure and suicide rates in the affected area, Ishikawa Prefecture, and nationwide are provided in ESM 4. These tables offer additional context and support for the findings discussed in this study.

Information on postdisaster public health and mental health countermeasures is also provided in ESM 4.

The Noto Peninsula earthquake occurred on 1 January 2024, which was a nationally important time to celebrate the New Year. As a result, there was extensive information dissemination through television, the internet, and social media. This may have created momentum for support and strengthened social ties. For those who were socially isolated by the disaster, this emotional backing may have distanced them from suicidal thoughts. This trend was also observed in the Great East Japan Earthquake. *KIZUNA*, a Japanese watchword expressing human connection, was frequently reported in the media as a symbol of social solidarity, reminding people of compassion and mutual assistance (Gerster, 2019). Like past disasters, even in localized disasters such as the Noto Peninsula earthquake, KIZUNA through social media may have reduced the social isolation of the affected population and reduced suicide risk (see ESM 5).

However, there are objections to the pulling together effect and honeymoon phase. That is, the suicide rate may have decreased due to the disruption of access to means of suicide. It has been reported that the Kobe earthquake reduced the number of suicides by jumping due to the collapse of many high-rise buildings (Shioiri et al., 1999). It is possible that in the disaster area targeted in this study, people were forced to live in evacuation centers due to collapsed houses and disrupted roads, which may have restricted access to means of suicide that they would normally have had (see ESM 6).

Despite short-term reductions in suicide risk following the earthquake during the honeymoon phase, there is concern that suicide risk may gradually increase due to prolonged shelter living, changes in life circumstances such as temporary housing, and accumulated fatigue. A long-term increase in suicide rates was reported following the 2004 Niigata Chuetsu earthquake (magnitude 6.8), which measured the impact of the disaster in a local area (Hyodo et al., 2010).

This study highlights the need for ongoing postdisaster monitoring to understand the changing impact on mental health over time. Governments and local authorities should consider implementing real-time monitoring systems that can track mental health indicators in parallel with the impact of disasters.

The economic impact may also negatively affect medium- to long-term suicide risk. The number of bankruptcies in Ishikawa Prefecture in 2024 has risen compared to 2023, and accommodation cancellations are expected to increase, leading to a decline in tourism demand (Ishii et al., 2024). During the COVID-19 pandemic, the Japanese government implemented the Go To Travel campaign, a nationwide travel discount campaign aimed at stimulating regional economic recovery from the depression caused by the pandemic. This policy was introduced to support the tourism industry and boost regional economic activities during a phase of increased suicide risk (Ministry of Health, Labour and Welfare, 2020). In the current crisis, economic support measures in disaster areas should also be considered to prepare for a medium- to long-term increase in suicide risk.

### Limitations

Economic variables such as the number of company bankruptcies and the unemployment rate could not be added to the model used in this study. These economic variables are important in capturing the impact on suicide, and some studies have included them in their models (Anzai et al., 2021).

Furthermore, this study used the number of suicides as an outcome and did not analyze the number of suicide attempts or the incidence of depression. It is necessary to include these indicators in future analyses.

### Conclusion

The study employed two models, a Poisson regression model and a prophet model, to assess the number of suicides following disasters. Both models indicated a short-term reduction in suicides in the localized area of the Noto region. However, there is concern that mental health in disaster-affected areas may worsen in the future. The model used in this study could be valuable for the ongoing assessment of postdisaster mental health. Governments and local authorities need to develop comprehensive support measures for this crisis.

## Electronic Supplementary Material

The electronic supplementary material is available with the online version of the article at https://doi.org/10.1027/0227-5910/a001003

**ESM 1.** Location and damage overview of the 2024 Noto Peninsula Earthquake.

**ESM 2.** Model diagnostics for the Poisson regression analysis.

**ESM 3.** Additional analyses of predicted and observed numbers of suicides.

**ESM 4.** Mental health infrastructure, suicide rates, and post-disaster mental health measures.

**ESM 5.** Internet search trends related to disaster mental health.

**ESM 6.** Comparison of suicide rates by method before and after the 2024 Noto Peninsula Earthquake.

## Notes

**Open Science:** Open Data: The information needed to reproduce all of the reported results is available at https://www.mhlw.go.jp/stf/seisakunitsuite/bunya/0000140901.html (Ministry of Health, Labour and Welfare, 2024c).
