# Supplementary material for: Short-Term Preliminary Evaluation of Suicide Following the 2024 Noto Peninsula Earthquake in Japan Using Time Series Analysis
Source: Crisis. 2025 Apr 30;46(4):218–24. doi: 10.1027/0227-5910/a001003 (PMC12288478; doi:10.1027/0227-5910/a001003)
Supplement: Supplementary file 1 [file cri_46_4_218_esm1.pdf]

© 2025 The Author(s). Distributed as a Hogrefe OpenMind article under the license CC BY 4.0 (https://creativecommons.org/licenses/by/4.0)

Electronic Supplementary Material 1 for https://doi.org/10.1027/0227-5910/a001003

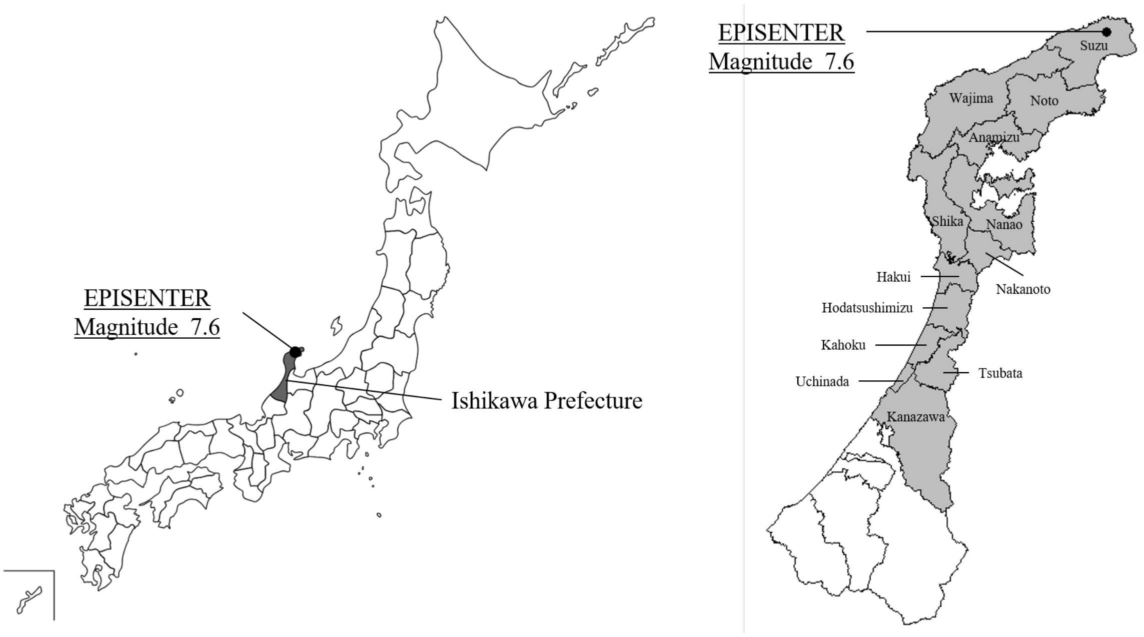

Figure E1. The Epicenter and Disaster Area of the 2024 Noto Peninsula Earthquake in Ishikawa Prefecture, Japan.

Table E1. Short-Term Casualties and Destroyed Homes in the Disaster Areas of 13 Municipalities Following the 2024 Noto Peninsula Earthquake.

	Population in January 2024	Households in January 2024	Death rate (/100,000)	Rate of wounded (/100,000)	Completely destroyed homes (/1,000household)	Half-completely destroyed homes (/1,000household)	Partially destroyed homes (/1,000household)
Kanazawa City	444667	213376	0.0	2.0	0.1	1.1	32.2
Nanao City	48015	21650	16.7	6.2	18.0	139.3	549.1
Wajima City	22839	11245	569.2	2259.3	359.4	427.3	530.9
Suzu City	12435	5784	916.8	2002.4	340.9	279.6	531.3
Hakui City	19831	8487	5.0	35.3	7.7	61.7	357.7
Kahoku City	36041	14333	0.0	0.0	0.6	17.0	188.7
Tsubata Town	37489	15003	0.0	5.3	0.6	5.3	171.2
Uchinada Town	26025	11339	0.0	19.2	10.8	48.3	129.2
Shika Town	18167	7839	11.0	572.5	70.4	306.4	566.1
Hodatsushimizu Town	11995	4881	0.0	0.0	2.5	15.0	314.7
Nakanoto Town	16794	6630	0.0	11.9	7.5	123.2	518.7
Anamizu Town	7283	3532	357.0	3528.8	140.7	406.0	596.5
Noto Town	15064	7154	119.5	331.9	33.1	122.9	626.1
Disaster area (Total)	1107528	499538	27.0	109.2	16.0	33.7	121.7
