# Supplementary material for: Short-Term Preliminary Evaluation of Suicide Following the 2024 Noto Peninsula Earthquake in Japan Using Time Series Analysis
Source: Crisis. 2025 Apr 30;46(4):218–24. doi: 10.1027/0227-5910/a001003 (PMC12288478; doi:10.1027/0227-5910/a001003)
Supplement: Supplementary file 2 [file cri_46_4_218_esm2.pdf]

© 2025 The Author(s). Distributed as a Hogrefe OpenMind article under the license CC BY 4.0 (https://creativecommons.org/licenses/by/4.0)

Electronic Supplementary Material 2 for https://doi.org/10.1027/0227-5910/a001003

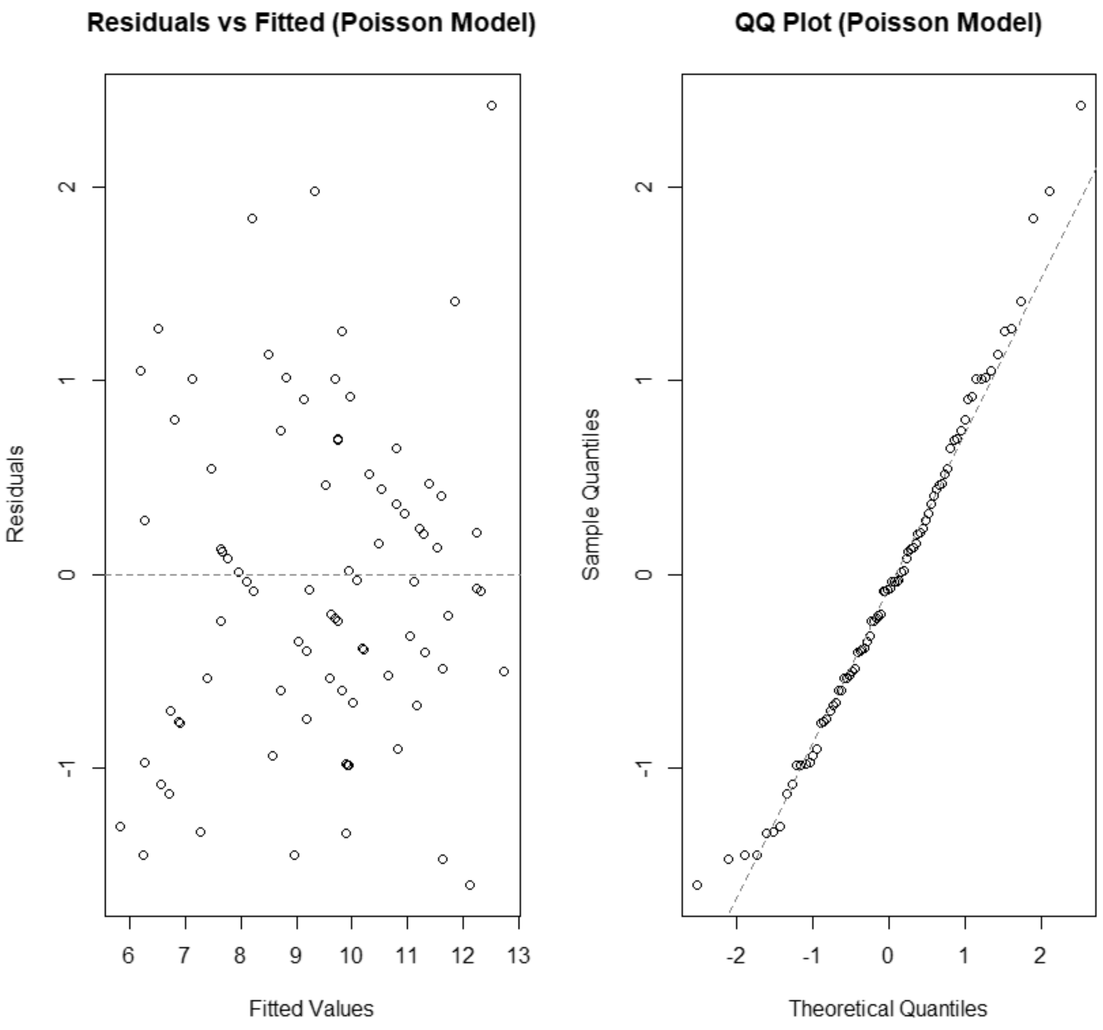

Figure E2. Residual plot (left) showing the residuals versus fitted values, and Q-Q plot (right) showing the quantiles of the residuals compared to a normal distribution for the Poisson regression model.

© 2025 The Author(s). Distributed as a Hogrefe OpenMind article under the license CC BY 4.0 (https://creativecommons.org/licenses/by/4.0)

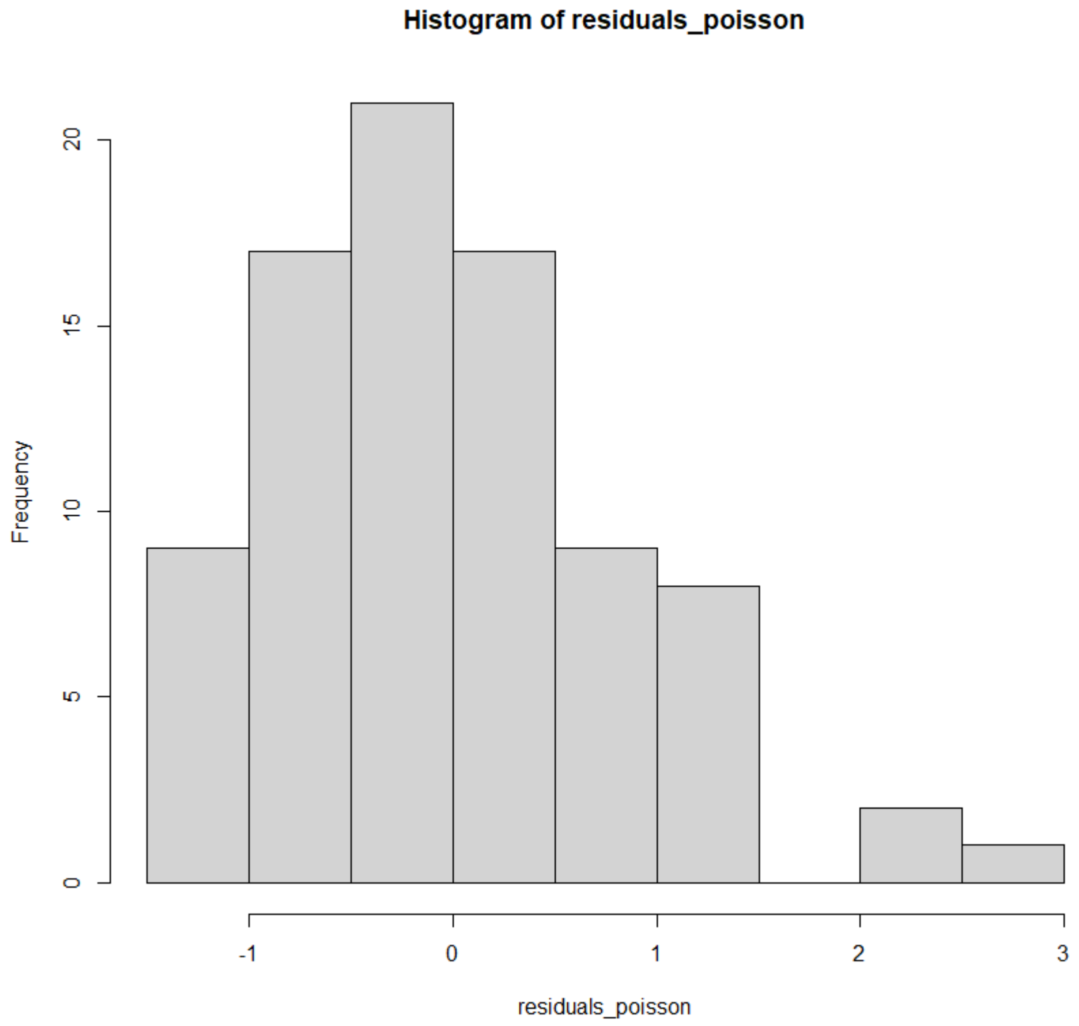

Figure E3. Histogram of Residuals for the Poisson regression model.

© 2025 The Author(s). Distributed as a Hogrefe OpenMind article under the license CC BY 4.0 (https://creativecommons.org/licenses/by/4.0)

Model Diagnostics
To assess the fit of the Poisson regression model, I used residual plots, QQ plots, and histograms. Additionally, I calculated the dispersion (variance-to-mean ratio). Based on these results, I evaluated the model's fit as follows:

Residual Plot
The residual plot shows the distribution of residuals against the predicted values (Figure S2, left). The residuals are distributed roughly randomly, without any systematic patterns. This indicates that the model does not exhibit bias and fits the data appropriately.

QQ Plot
The Q-Q plot evaluates whether the residuals follow a normal distribution (Figure S2, right). Most of the residuals lie along the 45-degree line, indicating they are close to a normal distribution. However, several points deviate from the 45-degree line, particularly at the tails. This suggests some deviation from normality but no major bias.

Residual Histogram
The residual histogram examines whether the residuals are symmetrically distributed (Figure S3). The residuals are approximately symmetrically distributed, though there is some skewness. This further suggests that the model fits the data reasonably well.

Dispersion (Variance-to-Mean Ratio)
The dispersion value is 0.89, which is less than 1. This indicates that there is no overdispersion problem. The absence of overdispersion means that the Poisson regression model is appropriate for the data.

Interpretation
These diagnostics provide confidence in the model's predictions and suggest that the Poisson regression model is appropriate for analyzing the monthly suicide counts in this study.
