# Supplementary material for: Short-Term Preliminary Evaluation of Suicide Following the 2024 Noto Peninsula Earthquake in Japan Using Time Series Analysis
Source: Crisis. 2025 Apr 30;46(4):218–24. doi: 10.1027/0227-5910/a001003 (PMC12288478; doi:10.1027/0227-5910/a001003)
Supplement: Supplementary file 3 [file cri_46_4_218_esm3.pdf]

© 2025 The Author(s). Distributed as a Hogrefe OpenMind article under the license CC BY 4.0 (https://creativecommons.org/licenses/by/4.0)

Electronic Supplementary Material 3 for https://doi.org/10.1027/0227-5910/a001003

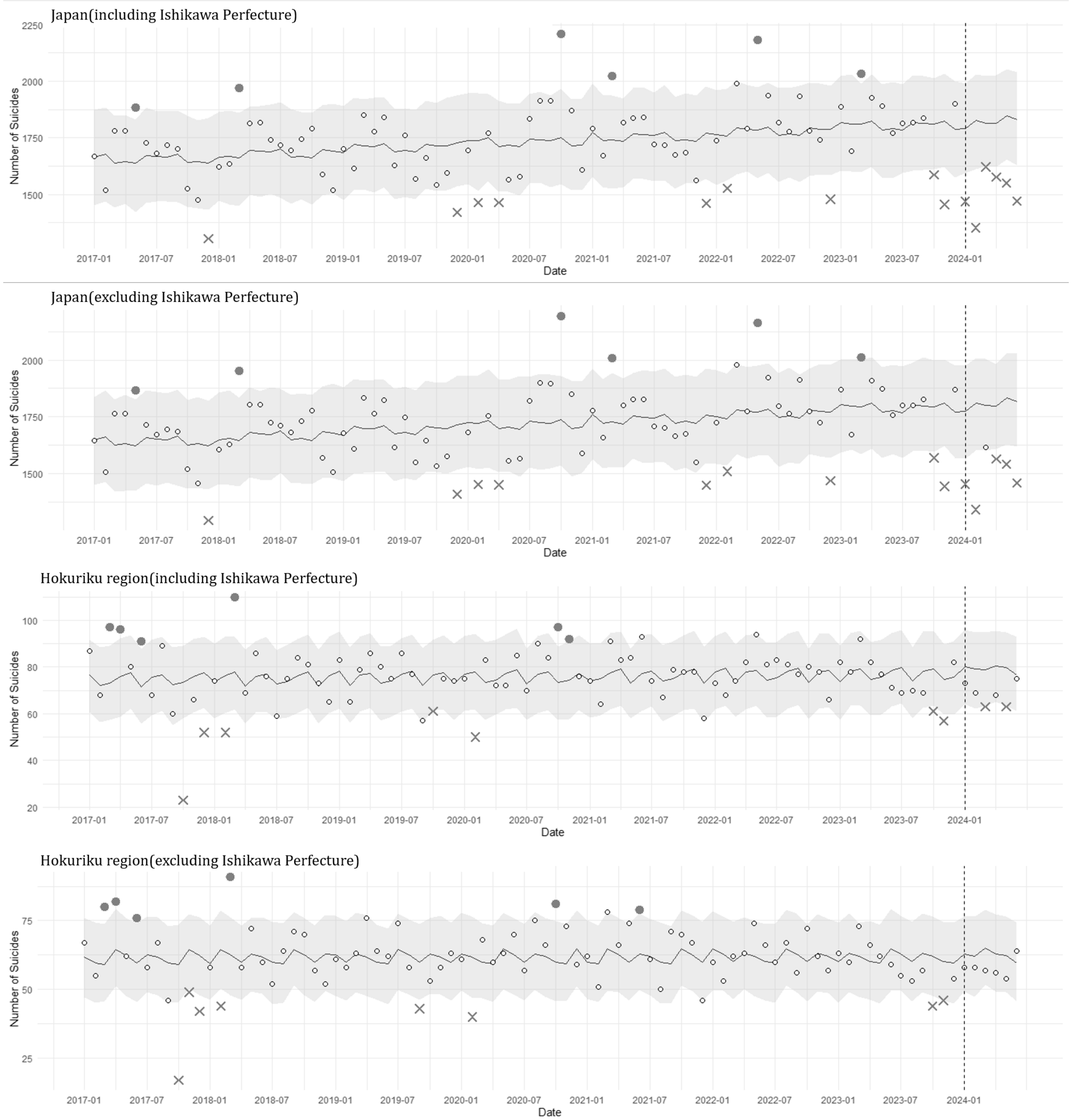

Figure E4. Plot of predicted and observed numbers of suicides for both Japan and the Hokuriku region (comprising Niigata, Toyama, Ishikawa, and Fukui prefectures), with and without Ishikawa Prefecture. Shaded areas represent confidence intervals. Excess suicides are indicated by filled bold circles, while fewer suicides than predicted are indicated by bold crosses.

© 2025 The Author(s). Distributed as a Hogrefe OpenMind article under the license CC BY 4.0 (https://creativecommons.org/licenses/by/4.0)

In this additional analysis, the Prophet model was used because the Poisson regression model assumes equidispersion, whereas the data exhibited substantial overdispersion, violating model assumptions and making it impractical.
Figure E4 shows that fewer suicides than predicted were observed in both Japan and the Hokuriku region when Ishikawa Prefecture was included after the earthquake.

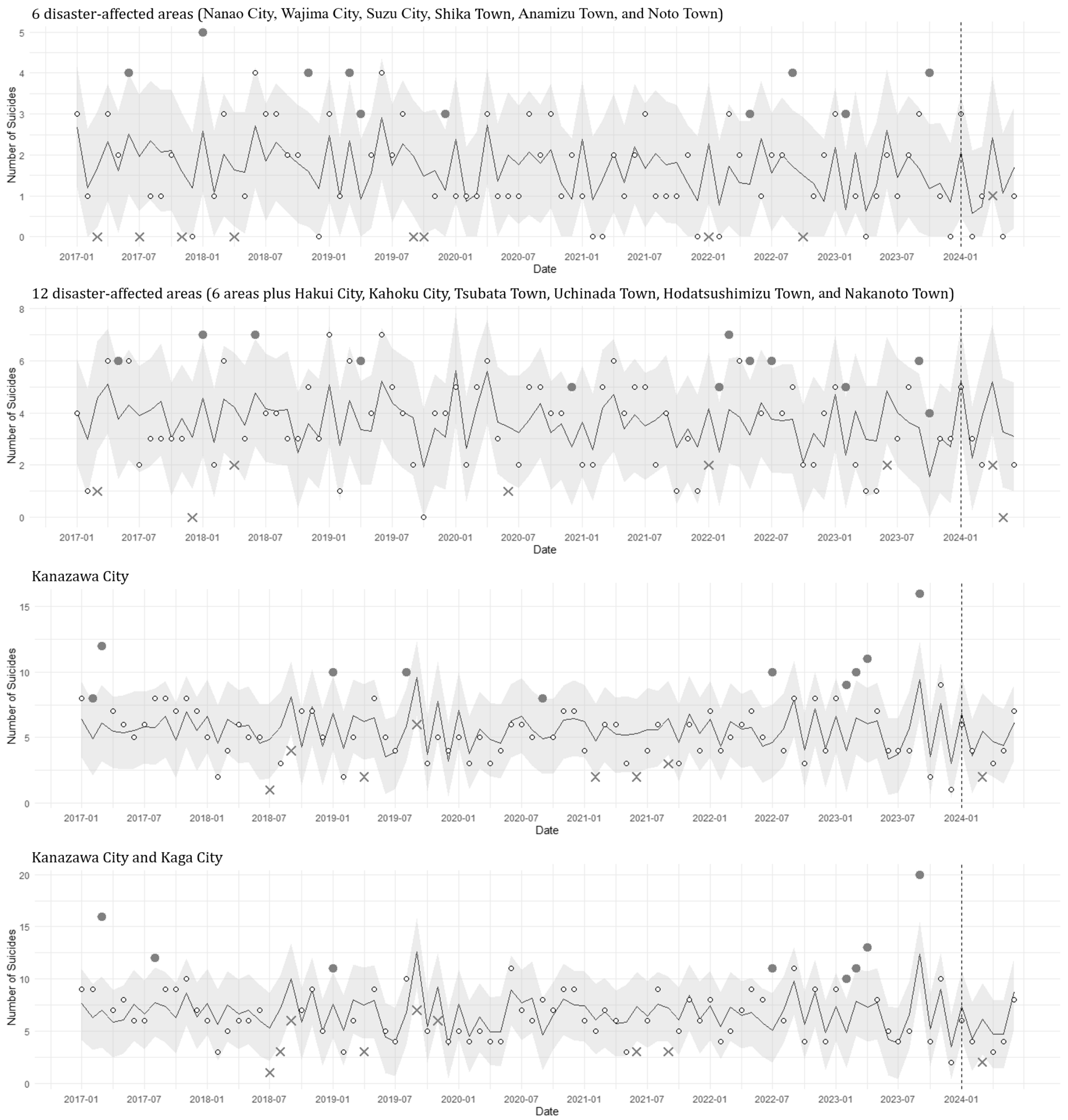

Figure E5. Plot of predicted and observed numbers of suicides for the six directly affected areas, 12 broader areas, Kanazawa City, and the combined area of Kanazawa City and Kaga City. Shaded areas represent confidence intervals. Excess suicides are indicated by filled bold circles, while fewer suicides than predicted are indicated by bold crosses.

© 2025 The Author(s). Distributed as a Hogrefe OpenMind article under the license CC BY 4.0 (https://creativecommons.org/licenses/by/4.0)

Additional analysis was conducted for the six areas that were directly affected by the earthquake (Nanao City, Wajima City, Suzu City, Shika Town, Anamizu Town, and Noto Town), as well as for the 12 areas, which include these six areas plus Hakui City, Kahoku City, Tsubata Town, Uchinada Town, Hodatsushimizu Town, and Nakanoto Town (all municipalities listed in Table 1 except for Kanazawa City). In addition, Kanazawa City, where economic damage was expected, and combined area of Kanazawa City and Kaga City were also analyzed using the Prophet model, bringing the total to four areas.

Figure E5 shows that fewer suicides than predicted were observed in April in the six directly affected areas, in April and May in the 12 areas, in March in Kanazawa City, and in March in the combined area of Kanazawa City and Kaga City.
