# Supplementary material for: Short-Term Preliminary Evaluation of Suicide Following the 2024 Noto Peninsula Earthquake in Japan Using Time Series Analysis
Source: Crisis. 2025 Apr 30;46(4):218–24. doi: 10.1027/0227-5910/a001003 (PMC12288478; doi:10.1027/0227-5910/a001003)
Supplement: Supplementary file 4 [file cri_46_4_218_esm4.pdf]

© 2025 The Author(s). Distributed as a Hogrefe OpenMind article under the license CC BY 4.0 (https://creativecommons.org/licenses/by/4.0)

Electronic Supplementary Material 4 for https://doi.org/10.1027/0227-5910/a001003

Table E2. Comparison of Mental Health Infrastructure Indicators in the Disaster Area, Ishikawa Prefecture, and Nationwide.

Indicator	Disaster area	Ishikawa Prefecture	Nationwide
Psychiatric inpatient institution	1.78	1.88	1.31
Psychiatric outpatient institution	1.00	4.11	6.80
Psychiatric medical institution	2.79	5.98	8.11
Public health center (main office)	0.45	0.45	0.37

Table E3. Annual Suicide Rates (per 100,000 Population) in the Disaster Area, Ishikawa Prefecture, and Nationwide (2017–2023)

Year	Disaster area	Ishikawa Prefecture	Nationwide
2017	18.07	16.73	16.52
2018	13.86	13.47	16.18
2019	15.26	14.4	15.67
2020	14.96	15.18	16.44
2021	12.08	13.07	16.44
2022	16.98	16.45	17.25
2023	17.26	17.72	17.27

The Mental Health and Welfare Infrastructure in the Disaster area

The affected area exhibited a relatively robust mental health and welfare infrastructure before the disaster, particularly in terms of medical institutions with psychiatric inpatient functions. According to the Ishikawa Prefecture Medical Plan (2024), the number of medical institutions with psychiatric inpatient functions in this administrative region was 1.78 per 100,000 population, comparable to the prefectural average of 1.88 and significantly higher than the national average of 1.31. However, the capacity of medical institutions with psychiatric outpatient functions was notably lower at 1.00 per 100,000 population, compared to 4.11 in Ishikawa Prefecture and 6.80 nationally.
The availability of psychiatric medical institutions overall followed a similar pattern, with 2.79 institutions per 100,000 population in the affected area, compared to 5.98

© 2025 The Author(s). Distributed as a Hogrefe OpenMind article under the license CC BY 4.0 (https://creativecommons.org/licenses/by/4.0)

in Ishikawa Prefecture and 8.11 nationally. Conversely, the number of public health centers (main offices) in the affected area was 0.45 per 100,000 population, identical to the prefectural average and slightly higher than the national figure of 0.37.
These indicators suggest that while the mental health and welfare system in the affected area was relatively strong overall, particularly in terms of inpatient services, the capacity for outpatient services was severely limited. Furthermore, as the figures for the affected area are based on the administrative divisions to which the area belongs, they may overestimate the actual availability of services in the disaster-affected areas. In reality, the system for outpatient medical institutions in these areas may be even more fragile than the data suggest.

Suicide Rate in the Disaster area

In terms of suicide risk, historical data from 2017 to 2023 indicate that suicide rates in the affected area were generally comparable to, or lower than, those in Ishikawa Prefecture and national averages. For example, in 2020, the suicide rate in the affected area was 14.96 per 100,000 population, slightly lower than the prefectural rate of 15.18 and the national average of 16.44. Across the seven years, fluctuations in suicide rates were observed, with the lowest rate recorded in 2021 (12.08) and the highest in 2017 (18.07). These trends suggest that the baseline suicide risk in the affected area was not significantly elevated compared to broader regional and national contexts prior to the disaster.

© 2025 The Author(s). Distributed as a Hogrefe OpenMind article under the license CC BY 4.0 (https://creativecommons.org/licenses/by/4.0)

Information on post-disaster public health and mental health countermeasures

After a disaster, survivors often experience significant psychological stress, making mental health support a critical component of intervention efforts. In response to the Kobe earthquake, Japan established Mental Health Care Centers to provide initial psychological support. These specialized teams have played a key role in stabilizing the mental health of disaster victims (Health and Global Policy Institute Mental Health Policy Project Team, 2022).

In the United States, the 24-hour Disaster Distress Helpline was launched following Hurricane Katrina to strengthen mental health support for disaster survivors (Substance Abuse and Mental Health Services Administration, 2024). Similarly, in New Zealand, the All Right? campaign was initiated after the 2011 Christchurch earthquake to promote mental health and foster community rebuilding. Such initiatives not only raise awareness about the mental health challenges faced by disaster victims but also contribute to strengthening community ties (Calder et al., 2022).

To formalize post-disaster mental health support, it is essential to establish a robust legal framework and design phased support plans (MHLW, 2021). However, top-down, state-led policies often result in delays in aid delivery. In contrast, grassroots, citizen-led initiatives can address survivors' feelings of social isolation and loneliness more quickly and sustainably (Gagné, 2020). To complement policy frameworks, it is important to explore participatory psychosocial support approaches through volunteer movements.

© 2025 The Author(s). Distributed as a Hogrefe OpenMind article under the license CC BY 4.0 (https://creativecommons.org/licenses/by/4.0)

References

Calder, K., Begg, A., D'Aeth, L., Turner, S., Fox, C., Nobes, B., Pope, K., & Bell, C. (2022). Evaluation of the all right? campaign for tangata whaiora/mental health service users in Canterbury, New Zealand. *Health Promotion International, 37*(1), Article daab102. https://doi.org/10.1093/heapro/daab102

Gagn´ e, I. (2020). Dislocation, social isolation, and the politics of recovery in post-disaster Japan. *Transcultural Psychiatry, 57*(5), 710–723. https://doi.org/10.1177/1363461520920348

Health and Global Policy Institute Mental Health Policy Project Team. (2022). *The past and future of mental health support during disasters in Japan: Reflections from regional disaster responses from 1995 to 2020*. Health and Global Policy Institute. https://hgpi.org/wp-content/uploads/JPN_Lessons-and-Future-Implications-of-Disaster-Mental-Health-Support-in-Japan_20221014.pdf

Substance Abuse and Mental Health Services Administration. (2024). SAMHSA – Substance Abuse and Mental Health Services Administration. U.S. Department of Health and Human Services. Retrieved December 1, 2024, from https://www.samhsa.gov/

Ministry of Health, Labour and Welfare. (2021). *Manual for mental health, medical, and welfare activities during disasters for local governments*. https://www.mhlw.go.jp/content/000772550.pdf
