# Supplementary material for: Short-Term Preliminary Evaluation of Suicide Following the 2024 Noto Peninsula Earthquake in Japan Using Time Series Analysis
Source: Crisis. 2025 Apr 30;46(4):218–24. doi: 10.1027/0227-5910/a001003 (PMC12288478; doi:10.1027/0227-5910/a001003)
Supplement: Supplementary file 5 [file cri_46_4_218_esm5.pdf]

**Electronic Supplementary Material 5 for [https://doi.org/10.1027/0227-5910/a001003](https://doi.org/10.1027/0227-5910/a001003)**

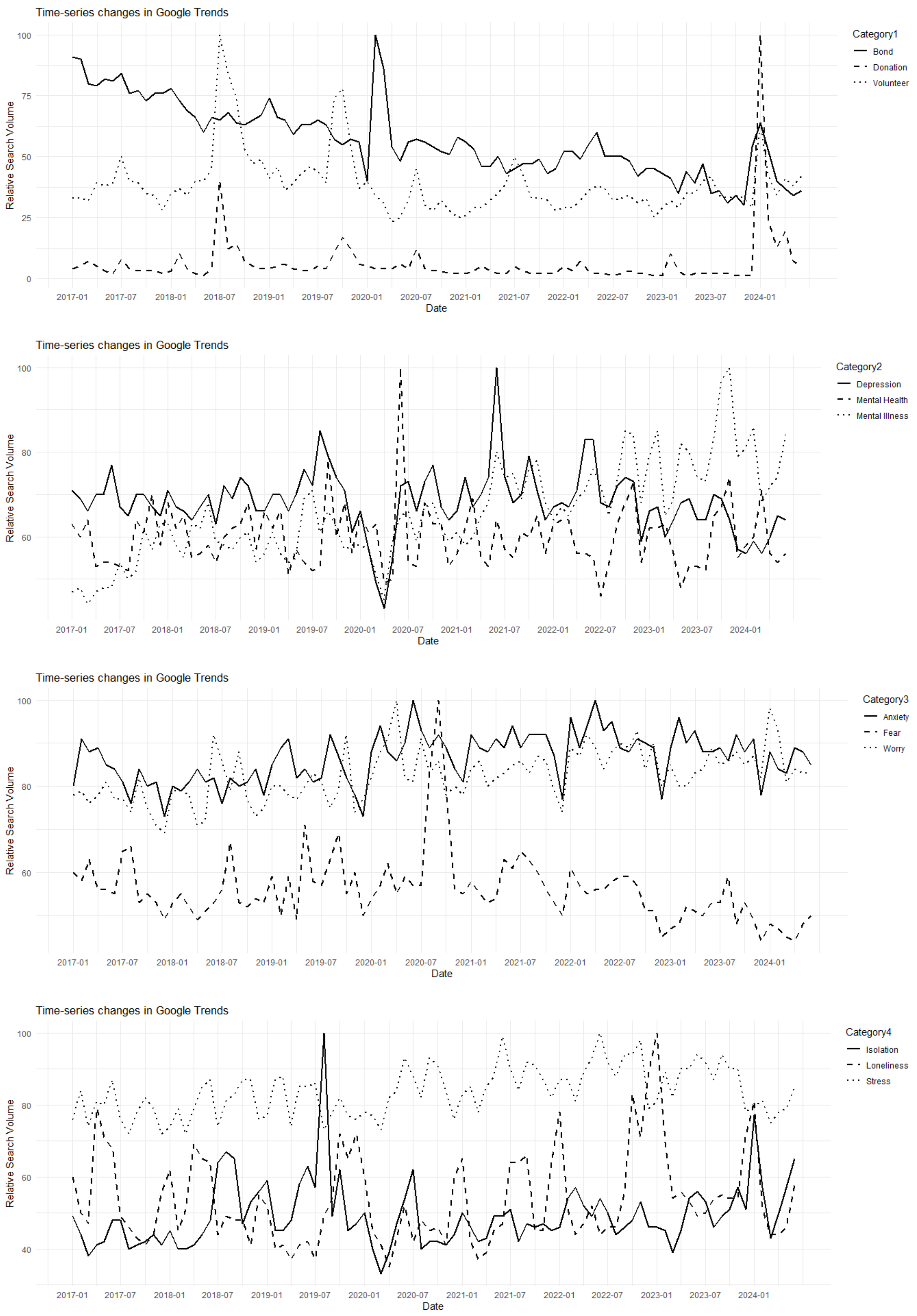


**Figure E6.** Internet searches (Relative Search Volumes) during the period of the survey (2017-01 to 2024-06).

© 2025 The Author(s). Distributed as a Hogrefe OpenMind article under the license CC BY 4.0 (https://creativecommons.org/licenses/by/4.0)

The change in the relative search volume (RSV) of these keywords over the survey period (January 2017 to June 2024) was analyzed.

The following representative search keywords have been selected for disaster mental health.
Category 1: "Bond (絆; kizuna)," "Volunteering (ボランティア; boranteia)," "Donation (義援金; gienkin)"
Category 2: "Depression (うつ病; utsubyou)," "Mental health (メンタルヘルス; mentaruherusu)," "Mental illness (精神疾患; seisinsikkan)"
Category 3: "Anxiety (不安; fuan)," "Worry (心配; shinpai)," "Fear (恐怖; kyoufu)"
Category 4: "Stress (ストレス; sutoresu)," "Loneliness (孤独; kodoku)," "Isolation (孤立; koritsu)"

The results suggest that RSVs for "Bond " "Volunteering," and "Donation" (Category 1) rose sharply immediately after the earthquake. Similarly, RSVs for "Worry," "Loneliness," and "Isolation" also showed notable increases. Searches for "Bond " reached a peak between January and February 2020, likely influenced by the COVID-19 pandemic, and again spiked in January 2024, immediately following the earthquake.
Among the analyzed keywords, "Donation" recorded the highest search volume during the study period, which could indicate a nationwide surge in support for disaster victims.
These search trends, particularly those related to "Bond" may serve as a useful tool for real-time monitoring of suicide risk following a disaster.

© 2025 The Author(s). Distributed as a Hogrefe OpenMind article under the license CC BY 4.0 (https://creativecommons.org/licenses/by/4.0)
