# Supplementary material for: Short-Term Preliminary Evaluation of Suicide Following the 2024 Noto Peninsula Earthquake in Japan Using Time Series Analysis
Source: Crisis. 2025 Apr 30;46(4):218–24. doi: 10.1027/0227-5910/a001003 (PMC12288478; doi:10.1027/0227-5910/a001003)
Supplement: Supplementary file 6 [file cri_46_4_218_esm6.pdf]

© 2025 The Author(s). Distributed as a Hogrefe OpenMind article under the license CC BY 4.0 (https://creativecommons.org/licenses/by/4.0)

Electronic Supplementary Material 6 for https://doi.org/10.1027/0227-5910/a001003

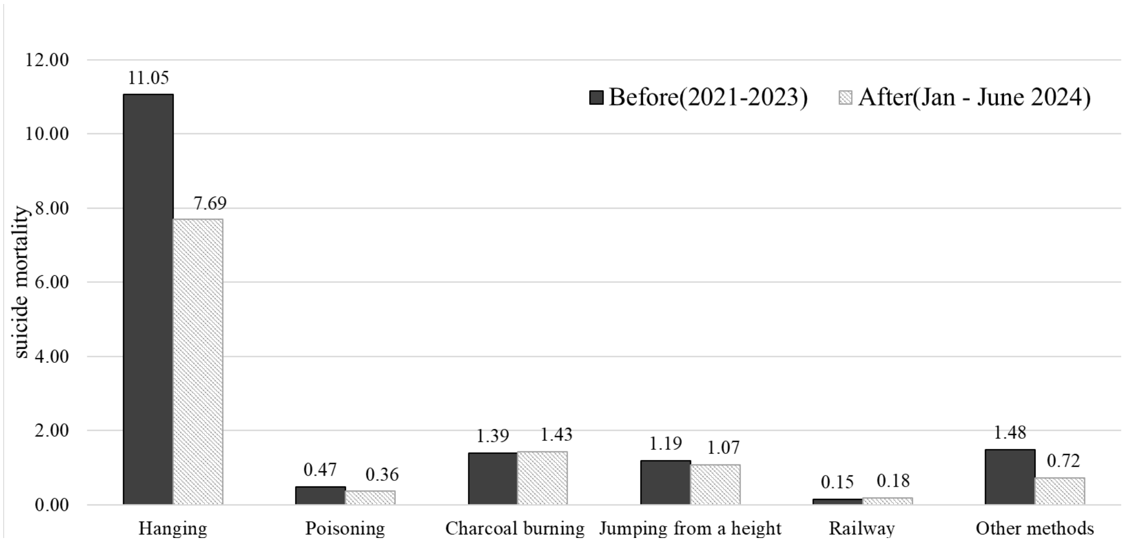

Figure E7. Comparison of suicide rates (by method) before and after the earthquake in Ishikawa Prefecture.

This analysis compared suicide mortality rates by method during the periods before the earthquake (2021-2023) and after the earthquake (January–June 2024).
The results revealed that not all methods of suicide decreased uniformly. Suicides by hanging decreased substantially from 11.05 to 7.69 per 100,000 population. This finding supports the possibility that the decrease in suicide rates after earthquakes may be explained by the restriction of specific means of suicide.
